# Assessing Personality Using Body Odor: Differences Between Children and Adults

**DOI:** 10.1007/s10919-013-0152-2

**Published:** 2013-04-16

**Authors:** Agnieszka Sorokowska

**Affiliations:** Institute of Psychology, University of Wroclaw, Ul. Dawida 1, 50-527 Wrocław, Poland

**Keywords:** Body odor, Olfaction, Personality assessment

## Abstract

Many studies have demonstrated that smell is an important sense in social interactions, and recently it was determined that olfactory cues might also convey information concerning certain personality traits. The present study investigated whether personality traits might be recognized using olfactory cues in contexts other than male–female interactions. 75 children (35 girls and 40 boys, *M* = 8.29 years) and 75 young adults (41 women and 34 men, *M* = 20.5 years) rated the personality traits of 50 unknown individuals (24 women and 26 men, *M* = 24.02 years) based on their body odor. The correlations between scent ratings and the self-assessed personality dimensions of odor donors were analyzed. The results show that both children and adults assess neuroticism relatively accurately, whereas only adults recognized dominance. These results suggest that olfaction supplements visual and auditory cues throughout our whole lives, contributing to the first impression accuracy of certain personality traits.

## Introduction

Body odor is an important variable in human social interactions. Although its significance has long been underestimated, many studies suggest that it plays a role in various aspects of our lives. For example, body odor might be involved in mate choice (e.g., by providing information about the genetic compatibility of a partner; Wedekind et al. 1995; Roberts et al. 2008), and the formation of early attachment between mothers and their babies (Winberg and Porter 1998). It could also indicate female fertility status (Havlicek et al. 2006; Singh and Bronstad 2001), and enable individual identification (Mallet and Schaal 1998; Olsson et al. 2006). A recent study (Sorokowska et al. 2012) suggested that, in addition to all of these functions, body odor might also convey information regarding the personality traits of neuroticism, dominance, and extraversion.

There exist some factors that influence body odor composition which also influence personality traits like extraversion, dominance, and neuroticism. Many studies suggest that the psychophysiological functioning of individuals is reflected in the levels of various chemical substances in their skin, saliva, urine, and genital secretions, all of which contribute to natural body odor (Kohl et al. 2001; Zouboulis 2004). Furthermore, hormones and neurotransmitters are related to these three traits (Carver and Miller 2006; Mazur and Booth 1998; Zuckerman 1995). McCrae and Costa (1990) suggest that conscientiousness, agreeableness, and openness to experience also have a biological basis, however, there seem to be no links between body odor and these traits. Overall, the biological parameters that influence body odors might also affect extraversion, dominance, and neuroticism; this is why these traits could be assessed based on body odors.

In addition, other lines of research might explain the link between certain personality dimensions (especially those which could be related to emotions, such as neuroticism or extraversion) and body odor. Human body odor is the result of bacterial action on the secretions of the eccrine, apocrine, and sebaceous glands (Jackman 1982), and psychological stimulation such as stress or excitement can activate the first two types of glands (Hurley and Shelley 1960; Sato 1977). Many studies suggest that our emotions influence body odor and the ways that others perceive us. Humans seem to be able to recognize fear (Ackerl et al. 2002; Chen and Haviland-Jones 2000) and happiness (Chen and Haviland-Jones 2000) using samples of sweat collected from the same individuals during different affective states. At the same time, neurotic people are prone to experiencing anxiety, guilt, or depression (McCrae and John 1992) and extraverted people are prone to being happier than average (Pavot et al. 1990).

Emotions are fundamentally ephemeral; however, if emotions experienced for a short time are detectable in body odor, and if people vary in the frequency of the specific emotions they experience due to individual differences, then it is likely that personality traits are associated with body odor through paths we still do not understand well. The axillary bacterial flora that produces body odor (Shelley et al. 1953) is most likely modified when organisms produce more or less secretions than usual; alternatively, its composition is specific due to repeated emotional experiences. Therefore, natural body odor might differ between people with neurotic traits and those who are emotionally stable, happy, and relaxed, like people of high extraversion and/or low neuroticism. Importantly, these differences might be salient enough for other people to detect them. One of the characteristics of human olfaction is the ability to learn quickly (Stevenson 2010). Furthermore, odorants immediately elicit associations and judgments (Savic and Berglund 2004). Thus, if people with neurotic tendencies or extraverts have a characteristic odor, then others might learn to judge the level of these traits based on scent.

The issue of assessing psychological traits based on body odor requires additional investigation. The results of previous studies (Havlicek et al. 2005; Rantala et al. 2006; Sorokowska et al. 2012) suggest that this ability might be limited to certain traits and specific male–female interactions. Havlicek et al. (2005) and Rantala et al. (2006) found evidence suggesting that women prefer the body odor of dominant males measured using a questionnaire completed by males in Havlicek et al. (2005) and the level of salivary cortisol in Rantala et al. (2006). Importantly, Havlicek et al. (2005) found that such preferences were the strongest in fertile women with a partner. Sorokowska et al. (2012) analyzed both same-sex and opposite-sex personality ratings based on body odor and found that the congruence between self- and other-assessed dominance was particularly high for ratings of the opposite sex and that women were particularly able to assess male neuroticism.

However, the relation between body odor and personality described above suggests that human olfactory communication might also be important in many contexts other than male–female interactions. Therefore, the ability to assess personality based on odor cues should emerge relatively early in human development; in fact, even prepubescent children demonstrate good sensitivity to social odors (for a review see Schaal 1988) and they should be able to judge others based on odor. Therefore, the current study examined whether prepubescent children, like adults, can assess certain personality traits in other people using only body odor.

## Method

This project consisted of two separate studies: one involving children and one involving young adults.

### Participants

The odor donors were the same 50 individuals (24 women and 26 men) aged between 18 and 30 years (*M* = 24.02; SD = 3.69) in both studies. In Study 1, the odor raters included 75 children (35 girls and 40 boys) aged between 7 and 9 years (*M* = 8.29; SD = 0.7). In Study 2, 75 young adults (41 women and 34 men) aged between 19 and 26 years (*M* = 20.5; SD = 1.22) assessed body odors. The experimenter recruited all volunteers—pupils and students from a few primary schools and two universities in Wroclaw, Poland (University of Wroclaw and Wroclaw University of Economics). All adult participants provided informed consent prior to their inclusion in the study. In the case of children, parental consent was obtained. The participants were not compensated for taking part in the study.

### Body Odor Sampling

Body odor samples were collected in the first phase of both studies. I decided to use only the body odors of adults because the apocrine glands that are the source of human pheromones (Thorne et al. 2002), and that are activated by emotions (Hurley and Shelley 1960) are not active before puberty. In addition, children do not have the axillary hairs that serve as a mechanical barrier for most chemicals produced there, including signaling chemicals (Shelley et al. 1953; Kohoutová et al. 2012).

Axillary cotton pads (rather than t-shirts, like in a previous study of odor-cue-based personality trait judgments, Sorokowska et al. 2012) were used to collect the odor samples. Cotton pads are preferred over t-shirts because confounding odorous substances are more likely to contaminate the latter (e.g., the smell of food or other people’s body odor). The significant odor substances related to personality traits should be present in both t-shirts and axillary pads.

The odor donors were provided with two experimental sets consisting of two 7 × 10 cm, 100 % cotton pads, surgical hypoallergenic tape, unscented soap, a sterile glass jar, and a new t-shirt. Donors washed themselves with the odorless soap the morning of the experiment day, then attached the cotton pads under their arms with the surgical tape, put on the provided t-shirt and wore the pads for 12 h during the day. The participants were asked to refrain from using scented cosmetics (e.g., fragrances, deodorants, and soaps), eating pungent foods (e.g., garlic, onions, or other spicy/odorous foods), and drinking alcohol or smoking starting the day prior to the experiment following the standard procedure of studies that involve body odor assessment (e.g., Havlicek et al. 2005). Procedural instructions were provided in person and on a special instruction sheet that also included a questionnaire concerning the individual’s activity during the testing period. No participant reported any major deviations from the procedure.

After 12 h, the participants placed the pads in the jars and returned them to the experimenter. The samples were then frozen overnight. (The freezing of the samples does not influence body odor quality, e.g., Lenochová et al. 2009; Roberts et al. 2008). The clearly defined procedure and instructions ensured that the samples differed minimally across both studies.

### Personality Assessment

After providing the body odor samples, donors completed a self-description personality questionnaire that consisted of six items (the same questionnaire was later completed by the child and adult raters). Five items were simplified versions of the Five Factor Model Scales (McCrae and Costa 1987): extraversion, agreeableness, conscientiousness, neuroticism, and openness to experience. The original bipolar items of the Big Five dimensions were not used because children needed to complete the questionnaire, and these items were too difficult. The last question concerned the self-perceived dominance of participants.

The Scale consisted of six following items, each consisting of three descriptors: (1) sociable, cheerful, and active (i.e., extraversion; E); (2) sympathetic, kind, and trusting (agreeableness; A); (3) dependable, self-disciplined, and responsible (conscientiousness; C); (4) anxious, nervous, and easily upset (neuroticism; N); (5) curious, likes changes, and has many new ideas (openness to experience; O); and (6) dominant, does not give in, and is a leader (dominance; D). Participants marked their answers on scale ranging from 1 to 5 (1 = *absolutely disagree*, 2 = *disagree somewhat*, 3 = *neither agree, nor disagree*, 4 = *agree* *somewhat*, and 5 = *absolutely agree*). Items were rated as a whole characterized by three descriptors.

The questionnaire was based on the Five Item Personality Inventory (FIPI; Gosling et al. 2003). In their original work, Gosling et al. (2003) defined each of the Big Five personality traits as two central and six additional descriptors (at least one of which was a negation). To make the items simpler and clearer, I chose only three descriptors and did not use negations. When choosing descriptors (or finding easier adjectives), I also used the narrative descriptions of the Big Five dimensions contained in the NEO-Five Factor Inventory manual (NEO-FFI, Costa and McCrae 1992; Polish adaptation by Zawadzki et al. 1998). The final list of characteristics was discussed with three 7-year-olds. Only the elements that were understandable to all children were retained in the questionnaire.

### Rating Sessions

The body odor samples were rated in the second phase of the study. Participants were told to imagine a person connected to the scent they smelled and rated his or her personality traits and sex on a paper-and-pencil questionnaire. Traits were rated on the same six 5-point dimensions upon which the donors had described themselves. Before the presentation of the odors, participants reported their potential olfactory deficits (e.g., rhinorrhea), and I excluded two children and one adult with olfactory problems, although other people were found to replace them. Each participant rated four randomly selected odor samples—at least one male and one female sample. Six raters (including at least two females and at least two males) assessed each odor sample.

The procedure was the same for children and young adults. However, the experimenter talked with the children about the procedure before rating the samples to assess whether they understood the idea of rating another person’s personality. In addition, the experimenter read the questionnaire to the children and evaluated whether they understood all of the characteristics they were to assess.

### Statistical Analyses

To assess the accuracy of sex assessments, the percentage of correctly assessed donor sex was calculated and compared with the 50 % accuracy expected by chance using a one-sample *t* test. The consensus across judges was evaluated using intraclass correlation coefficients (ICC 1, 6; Shrout and Fleiss 1979). Spearman’s rank correlation was used to assess the agreement between the average ratings based on scent for all the judges and the self-assessed personality dimensions of the odor donors. Correlations were computed separately for adults assessing all female and male targets, as well as for children assessing all female and male targets. Nonparametric testing was used because the distribution of self-assessed personality traits was not normal; moreover, the rating scale was ordinal.

The descriptive statistics for self-assessed personality traits of odor donors were as follows: extraversion, *M* = 3.63, SD = 1.49; agreeableness, *M* = 3.51, SD = 1.77; conscientiousness, *M* = 3.37, SD = 1.07; neuroticism, *M* = 2.71, SD = 1.18; openness to experience, *M* = 3.10, SD = 1.39; and dominance, *M* = 3.30, SD = 1.14. The mean values for all averaged assessments by children were as follows: extraversion, *M* = 3.19, SD = 0.60; agreeableness, *M* = 3.29, SD = 0.48; conscientiousness, *M* = 3.20, SD = 0.51; neuroticism, *M* = 2.80, SD = 0.73; openness to experience, *M* = 3.20, SD = 0.62; and dominance, *M* = 2.83, SD = 0.61. The mean values for all averaged assessments by young adults were as follows: extraversion, *M* = 3.28, SD = 0.63; agreeableness, *M* = 3.17, SD = 0.60; conscientiousness, *M* = 3.45, SD = 0.51; neuroticism, *M* = 3.09, SD = 0.64; openness to experience, *M* = 3.03, SD = 0.73; and dominance, *M* = 3.16, SD = 0.60.

## Results

According to the Shapiro–Wilk test of normality, none of the self-assessed traits were normally distributed (all *p* < .05).

### Children

Target sex was identified correctly in 51 % of the ratings across all scent assessments, which was not significantly above chance, *t* (49) = .47, *p* = .46.

The mean consensus (ICC 1, 6) for all analyzed dimensions was .18. Consensus was significant only for neuroticism (ICC = .35, *p* = .031). The ratings of extraversion (ICC = .09, *p* = .33), agreeableness (ICC = .09, *p* = .33), openness (ICC = .11, *p* = .33), conscientiousness (ICC = .10, *p* = .31), and dominance were not consistent (ICC = .08, *p* = .35).

**Fig. 1:**
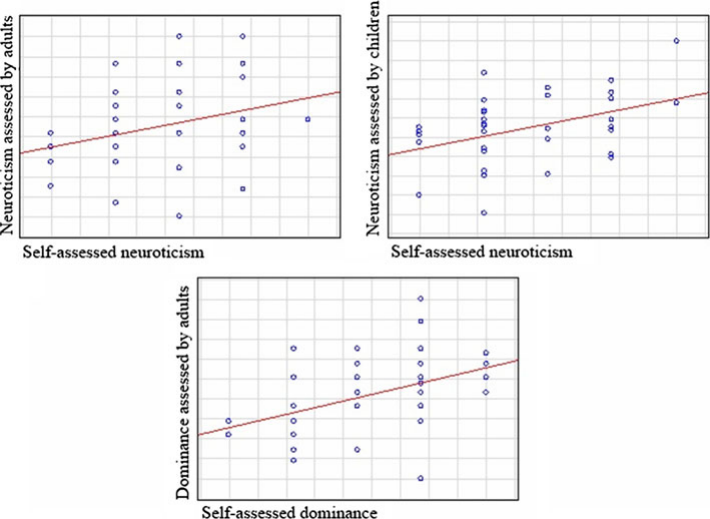
Significant correlations between self-reported donor traits (neuroticism and dominance) and child/adult judgments based on scent

With regard to child judgments of all donors, self-other agreement was significant only for neuroticism (*rs* = .37; *p* = .008). In addition, when male and female target assessments were analyzed separately, self-other agreement with regard to neuroticism was only significant for male targets (*rs* = .48; *p* = .01; see Fig. 1). All results are presented in Table 1.

**Table 1:** Spearman’s rank correlations between self-reported donor traits and child/adult judgments based on scent

Trait	Children rating all donors (*N* = 50)	Children rating women (*n* = 24)	Children rating men (*n* = 26)	Adults rating all donors (*N* = 50)	Adults rating women (*n* = 24)	Adults rating men (*n* = 26)
	*rs*	*rs*	*rs*	*rs*	*rs*	*rs*
Neuroticism	.37**	.17	.48*	.32*	.41*	.28
Extraversion	.10	.21	−.09	−.04	.18	−.25
Openness to experience	.06	.22	−.21	.10	.16	.17
Conscientiousness	.05	−.08	.09	−.06	−.13	−.06
Agreeableness	.12	−.04	.18	−.08	−.09	−.08
Dominance	−.02	−.17	.10	.42**	.44*	.39*

Two-tailed test

* *p* < .05, ** *p* < .01

### Young Adults

In all scent assessments, the target sex was identified correctly in 60 % of ratings, which was significantly above chance, *t* (49) = 3.15, *p* = .003.

The mean consensus coefficient (ICC 1, 6) for all the analyzed dimensions was .51. The consensus for specific dimensions was significant, and the highest ICCs were for neuroticism, extraversion and agreeableness (ICC = .57, *p* < .001), followed by openness to experience (ICC = .52, *p* < .001), conscientiousness (ICC = .47, *p* = .03), and dominance (ICC = .35, *p* = .034).

Self-other agreement was significant and highest for neuroticism (*rs* = .32; *p* < .05) and dominance (*rs* = .42; *p* = .002) (see Fig. 1). All targets judgments concerning the other dimensions (extraversion, openness to experience, conscientiousness, and agreeableness) were not significant. In addition, when assessments of male and female targets were analyzed separately, self-other agreement was significant for neuroticism in females (*rs* = .41; *p* < .05) as well as dominance in females (*rs* = .44; *p* = .03) and males (*rs* = .39; *p* < .05). All results are presented in Table 1.

## Discussion

In this study I found that children and adults can assess neuroticism (i.e., the enduring tendency to experience anxiety, depression, nervousness, and instability, McCrae and Costa 1987; McCrae and John 1992) by smelling. Among adults, I also found a positive correlation between self-assessed dominance and how others judged a person’s dominance based on the person’s odor. I conclude that the ability to assess personality traits using body odor goes beyond mating and that people seem to acquire this competence in childhood.

Among the personality traits studied, subjects rated neuroticism most consistently. My findings beg two questions. First, why do we find a positive correlation between neuroticism and body odor? Second, why were child judgments of neuroticism on the basis of body odor congruent with self-assessed neuroticism of odor donors? I next explore three possible answers to the two questions.

First, studies (Ackerl et al. 2002; Albrecht et al. 2011; Chen and Haviland-Jones 2000; Chen et al. 2006) suggest that fear and stress affect the production of substances, which, in turn, bear an association with body odor and that neurotic people are inclined to fear and stress (McCrae and John 1992). Recall from the section entitled *Personality assessment*, that I defined neuroticism as a mixture of anxiousness, nervousness, and being easily upset. Therefore, if people distinguished between (i) sweat samples collected from people who experienced fear and (ii) sweat samples collected from people who experienced neutral emotions over a short time (e.g., 70 min in Ackerl et al. 2002), it follows that they could also assess neuroticism based on the sweat samples of people who wore axillary pads over a full day during which they most likely experienced fear and stress. In addition, prolonged exposure to such feelings might also change the odor of donors over the long term. For example, repeated, emotionally induced sweating might modify the bacterial flora in the axillae, causing neurotics to smell differently.

Second, people could accurately assess neuroticism based on odor cues because neuroticism and human physiology overlap (Zuckerman 1995). Anxiety-related traits, such as neuroticism, correlate with low serotonin turnover (Carver and Miller 2006; Reif and Lesch 2003), and serotonin contributes to human physiological functions, such as food intake and mechanisms which regulate eccrine sweating intensity and several psychological disorders (Carver and Miller 2006; Stahl et al. 1983). Thus, biological processes which influence body scent and neuroticism levels might make odor a cue for neuroticism.

Last, child judgments of neuroticism on the basis of body odor can be congruent with self-assessed neuroticism of odor donors because of the effects that parental neuroticism has on children. For example, Engfer and Schneewind (1982) suggest that the emotional instability of parents contributes to harsh parental punishment. This finding might explain why children quickly learn to recognize emotional instability, and why they use all available cues, including olfactory information, to assess neuroticism. It is unclear why children judged neuroticism more accurately in males than in females, and why adults judged neuroticism more accurately in females than in males.

Why did adults but not children rate dominance accurately? Dominant and aggressive behaviors tend to correlate positively with elevated levels of testosterone and its metabolites (Cashdan 1995; Gray et al. 1991). In addition, testosterone stimulates the proliferation of sebocytes and affects the functioning of the apocrine sweat glands (Zouboulis 2004). Dominant people might smell differently than submissive people; therefore, children should be able to assess dominance based on body odor. Children’s olfactory abilities probably do not explain fully their incorrect answers. Although the olfaction of children improves with age both in the case of kin recognition (Ferdenzi et al. 2009) and other odors (Schaal 1988), olfaction is well-developed, including sensitivity to certain body odor compounds such as androstenes (Dorries et al. 1989). In summary, the olfactory abilities of children resemble those of young adults and, if the scent of dominance exists, children should be able smell it. However, they were unable to do so in my study.

One reason why children did not assess the dominance level based on body odor is that other people’s dominance (and the ability to assess it correctly) might become important only after puberty, especially in mating. Studies suggest that women in their fertile phase of their reproductive cycle liked most the scent of dominant men (Havlicek et al. 2005), and that judgments of dominance were most accurate when participants rated the odor of the opposite sex (Sorokowska et al. 2012). Dominance correlates positively with testosterone (Mazur and Booth 1998), and can suggest that the person is healthy and fertile. Consequently, people might learn to assess dominance using body odor only after mate choice becomes important.

Dominance is related to aggressiveness, and detection of aggressiveness might be meaningful for a child when dealing with adults, but dominance that the children assessed did not include aggressiveness. The children might have assessed the dominance of odor donors more accurately had the relation between dominance and aggression been more clearly indicated in the questionnaire. In addition, the age of the odor donors could influence the assessments—children might better assess their peers than the adults.

Contrary to findings from a previous study (Sorokowska et al. 2012), participants did not rate extraversion accurately. At least two reasons might explain the finding. First, this study included a slightly different personality characteristic. Here, I defined an extrovert as a sociable, cheerful, and active person, and in the previous study (Sorokowska et al. 2012) we used the full NEO-FFI Scale, which measured also elements such as assertiveness and enthusiasm. Maybe these (or some other) excluded elements of extraversion mattered for assessments of extraversion using body odor. On the contrary, it seems that “anxious, nervous, and easily upset” or “dominant, does not give in, and is a leader” used my current study were enough to judge neuroticism and dominance, respectively, using olfactory cues. I suggest that researchers in future studies should analyze whether different questionnaires (i.e., standardized inventories or self-assessment adjective scales) affect the assessments based on smelling.

Second, the participants could have rated extraversion inaccurately because the cotton pads did not convey well information about extraversion. In general, results from cotton pads and t-shirts should not differ in experiments that rely on personality assessments using smelling. Axillae contain bacteria which generate body odor (Leyden et al. 1981), and glands influence by emotional experiences (Hurley and Shelley 1960; Sato 1977), androgens, and pituitary hormones (Thody and Shuster 1989). Nevertheless, the heterogeneous surface of skin covered by a t-shirt probably produces additional odors and creates more complex odor sample than those of axillary pads. The level of extraversion might correspond with a person’s hygiene or food habits, producing odorous environmental contaminations which might add information to the final scent of the t-shirt. Researchers in future studies could study the influence of different procedures (i.e., t-shirts vs. cotton pads) on assessments of personality using body odor.

It is still unclear why the participants did not assessed openness to experience, conscientiousness, and agreeableness accurately using body odor. Both in the present study and Sorokowska et al. (2012) judgments of agreeableness, openness to experience, and conscientiousness were not congruent with the self-assessed traits of odor donors. However, the finding that adult raters agreed on their personality evaluations (according to the ICCs) suggests that they might have based their judgments on a given characteristic of the odor. Thus, this common factor might be very weakly related (or not related at all) to the evaluated personality trait; alternatively, people might not have the ability to correctly infer personality traits based on odors of substances related to these traits. Additional information might be needed to accurately assess conscientiousness, agreeableness, and openness to experience (e.g., social context or longer acquaintanceships). However, further investigations are necessary to determine whether this supposition is correct.

The present study has some limitations. First, children assessed the body odor samples of adults. Additional analyses should be conducted to determine whether their judgments are more congruent when assessing their peers. However, children have weaker body odor, and the procedure of such a study would have to be modified.

The exact mechanism that allows people to assess personality traits based on body odor still must be investigated. However, the present study demonstrated that congruent personality judgments based on olfactory cues develop to some extent during childhood. Although some of the obtained results are difficult to explain at this stage of research, the present study expands the understanding of personality perception in both children and adults. The results show that human body odor might convey important information regarding personality traits, and olfaction may supplement visual and auditory cues, thereby contributing to the accuracy of first impressions.
